# The influence of Achilles tendon mechanical behaviour on “apparent” efficiency during running at different speeds

**DOI:** 10.1007/s00421-020-04472-9

**Published:** 2020-08-25

**Authors:** Andrea Monte, Constantinos Maganaris, Vasilios Baltzopoulos, Paola Zamparo

**Affiliations:** 1 Department of Neurosciences, Biomedicine and Movement Sciences, University of Verona, via Felice Casorati, 43, 37131 Verona, Italy; 2 Research Institute for Sport and Exercise Sciences (RISES), Liverpool John Moores University, Liverpool, UK

**Keywords:** Running efficiency, Tendon mechanics, Elastic energy, Gastrocnemius medialis

## Abstract

**Purpose:**

We investigated the role of elastic strain energy on the “apparent” efficiency of locomotion (AE), a parameter that is known to increase as a function of running speed (up to 0.5–0.7) well above the values of “pure” muscle efficiency (about 0.25–0.30).

**Methods:**

In vivo ultrasound measurements of the gastrocnemius medialis (GM) muscle–tendon unit (MTU) were combined with kinematic, kinetic and metabolic measurements to investigate the possible influence of the Achilles tendon mechanical behaviour on the mechanics (total mechanical work, *W*_TOT_) and energetics (net energy cost, *C*_net_) of running at different speeds (10, 13 and 16 km h^−1^); AE was calculated as *W*_TOT_/*C*_net_.

**Results:**

GM fascicles shortened during the entire stance phase, the more so the higher the speed, but the majority of the MTU displacement was accommodated by the Achilles tendon. Tendon strain and recoil increased as a function of running speed (*P* < 0.01 and *P* < 0.001, respectively). The contribution of elastic energy to the positive work generated by the MTU also increased with speed (from 0.09 to 0.16 J kg^−1^ m^−1^). Significant negative correlations (*P* < 0.01) were observed between tendon work and metabolic energy at each running speed (the higher the tendon work the lower the metabolic demand) and significant positive correlations were observed between tendon work and AE (*P* < 0.001) at each running speed (the higher the tendon work the higher the efficiency).

**Conclusion:**

These results support the notion that the dynamic function of tendons is integral in reducing energy expenditure and increasing the “apparent” efficiency of running.

## Introduction

Human locomotion entails the motion of the body through an environment: air in terrestrial locomotion whilst in contact with the ground, and water in aquatic locomotion. The minimum required work that has to be done to maintain the motion of any object in its surrounding environment, is given by the product of the resistance offered by the environment and the distance covered during the motion. The efficiency of the locomotor apparatus can thus be expressed as the ratio between the work necessary to maintain motion and the chemical energy transformed by the muscles. This “locomotion” efficiency (i.e. the total mechanical work generated at whole-body level as a proportion of metabolic cost), has been investigated in several forms of terrestrial and aquatic locomotion, such as swimming (e.g. Zamparo et al. 2002), cycling (e.g. Minetti et al. 2001), walking and running (e.g. Cavagna and Kaneko 1977; Lejeune et al. 1988; Williams and Cavanagh 1987).

In the above-mentioned studies, “locomotion” efficiency was, actually, calculated as the ratio between (total) mechanical work per unit distance (*W*_TOT_) and net energy cost (*C*_net_, the metabolic energy expended per unit distance); in turn, *C*_net_ was calculated as the ratio between net oxygen uptake and locomotion velocity ($\dot{V}$O_2net_/*v*) and *W*_TOT_ was calculated as the sum of two components: *W*_EXT_ (the work done to raise and accelerate the body centre of mass within the environment) and *W*_INT_ (the work associated with the acceleration of body segments with respect to the centre of mass). As calculated, “locomotion” efficiency approximates “pure” muscle efficiency values (about 0.25–0.30, as reported by Woledge et al. 1985) in the forms of locomotion where elastic recoil is negligible [e.g. swimming or cycling, as reported by Zamparo et al. (2002) and Minetti et al. (2001)] whereas in the case of running, the efficiency calculated in this manner can reach far larger values [e.g. up to 0.5–0.7, as reported by Cavagna and Kaneko (1977)].

“Locomotion” efficiency is, therefore, often referred to as “apparent” efficiency because an increase beyond pure muscle efficiency values does not indicate that the muscles work in a more efficient way (Ettema 2001). Rather, these increased efficiency values are an indication of the conversion of metabolic energy into mechanical work at whole-body level. As suggested by Alexander (1991), measuring “apparent” efficiency can thus help in understanding whether mechanical work is “recycled” via storage and release of elastic energy (an energy saving mechanism).

As an example, when running at steady-state speed, tendons stretch and recoil; through this succession of stretch–shortening cycles, tendons could play an important role as energy savers allowing this form of locomotion to be particularly efficient (Roberts and Azizi 2011). In these conditions, indeed, “apparent” efficiency (AE = *W*_TOT_/*C*_net_) increases linearly with speed because *W*_TOT_ increases whereas *C*_net_ does not show appreciable changes when the velocity increases (e.g. Cavagna and Kaneko 1977). In other conditions (e.g. shuttle running or uphill running), AE is much lower and this could be attributed to the fact that, in these conditions, the tendon acts more as a power amplifier (Roberts and Azizi 2011). As an example, during shuttle running, AE is lowest over short shuttle distances covered at maximal speed (e.g. when the accelerations and decelerations are larger and the tendons’ capability to save metabolic energy is expected to be reduced) and highest over long shuttle distances (e.g. in conditions that approximate those of constant speed, linear, running where the metabolic energy saving mechanism is expected to be more prominent) (Zamparo et al. 2019). A further example is that of running on sand where AE is lower relatively to running on a hard surface and this could be attributable to a decrease in “muscle–tendon efficiency”, the sand acting as a damper which reduces the energy that can be recoiled from the stretched tendon (Lejeune et al. 1988). Taken together, these findings suggest a link between the capability to exploit the elastic energy mechanisms in tendons and the values of “apparent” efficiency in human running.

The dynamic function of tendons is indeed integral to reduce the energy expenditure of steady-state running: energetic savings may occur by shifting the operating regions of the muscles on their force–length and force–velocity curves (Ramsey and Street 1940), by reducing muscle work (Biewener and Roberts 2000), or by reducing active muscle volume (Holt et al. 2016). As an example, an active muscle uses less metabolic energy and produces more force when operating under isometric conditions compared to shortening (Fenn 1924). In addition, if the muscle operates close to optimal length (the length corresponding to optimum myofilament overlap) it will produce more force (Gordon et al. 1966) for a given activation level. Therefore, a quasi-isometric behaviour (i.e. slow shortening speed) around optimal muscle length enables the muscle to produce high forces more economically. During different forms of locomotion (e.g. walking and running), the elastic elements could accommodate the largest part of the MTU length changes, allowing the fascicles to work at a high force–length–velocity potential (Fukunaga et al. 2001; Lichtwark et al. 2007; Bohm et al. 2019; Monte et al. 2020). Without tendons, the fascicle shortening velocity would be higher, increasing the cross-bridge turnover and the energy demand for muscle contraction (Woledge et al. 1985). Furthermore, since the force per cross-bridge decreases with increasing velocity (de Tombe and Ter Keurs 1990), a decrease in the muscle force potential would require an increased muscle activation to maintain the same level of force to support and accelerate the body’s centre of mass, thereby increasing the energy cost of locomotion (Fletcher and MacInthos 2017). Based on these theoretical considerations, if the whole muscle–tendon unit length changes could be attributed to the tendon only, the muscle fibres would operate under isometric conditions, reducing the fascicle length changes (and, therefore, the mechanical work done by the muscles) and the level of muscle activation required for a given force (Fletcher and MacInthos 2017). These theoretical notions were supported by recent studies of Bohm et al. (2019) and Monte et al. (2020), which revealed that the plantar flexor muscles operated quasi-isometrically at a high-force potential during running at different speeds. Furthermore, Bohm and co-workers found a negative significant correlation between the force–length–velocity potential of the soleus muscle and the energy cost of running at 10 km h^−1^, suggesting that the higher the force potential the lower the energy expended. Therefore, the ankle plantar flexors seem to play an important role in the mechanical and physiological demand of human running.

The human ankle plantar flexors produce forces of up to 12 times body weight during running at increasing speed (Komi 1990) and are the main force producers amongst all the major lower limb muscle groups (Dorn et al. 2012). Due to their unique design (short muscle fibres connected to the heel via a long and compliant Achilles tendon), the ankle plantar flexors, have the capacity to generate high amounts of power with minimal energy expenditure. Thanks to their long tendon, the plantar flexors can store elastic energy up to about 60% of the MTU mechanical work during running (Monte et al. 2020; Farris and Sawicki 2012; Lai et al. 2014) and their contribution increases as a function of speed. This behaviour seems to be particularly relevant for determining energy expenditure, mechanical work and, therefore, “apparent” efficiency; however, there are no studies that have investigated the role of plantar flexor tendons on “apparent” running efficiency.

The aim of this study was to verify experimentally the theoretical link between human plantar flexor muscle–tendon behaviour and “apparent” running efficiency. In particular, we combined in vivo ultrasound, kinematic and kinetic measurements during running at different speeds and we calculated the relative contribution of GM muscle fascicles and Achilles tendon to the mechanical work done by the MTU to investigate the role of Achilles tendon behaviour on the mechanical power output at whole-body level (*W*_TOT_) and on the energy demands ($\dot{V}$O_2net_ and *C*_net_) during running at increasing speeds. Our main hypothesis was that the contribution of tendon work to the total work done by the MTU would increase with running speed and that this increase could, at least partially, explain the concurrent increase in “apparent” efficiency.

## Materials and methods

### Ethical approval

All participants received written and oral information and instructions before the study and gave their written informed consent to the experimental procedure. The experimental protocol was approved by the Ethical Committee of Liverpool John Moores University (protocol number: 18/SPS/028) and was performed in accordance with the Helsinki Declaration.

### Participants

The experiments were performed on 15 male endurance athletes, as a part of a larger study (Monte et al. 2020). All participants (24 ± 2.4 years of age; 74 ± 2.8 kg of body mass; 1.77 ± 0.04 m of stature; 8.5 ± 2.2 years of training: 5 ± 1 workouts per week) received written and oral instructions before the study and gave their written informed consent to participate in the experimental procedures. The experimental protocol was approved by the Ethical Committee of Liverpool John Moores University (protocol number: 18/SPS/028) and performed in accordance with the Helsinki Declaration.

### Experimental design

During each running trial, the participants ran at steady-state speed using a self-selected cadence, step length and running technique. All participants used a forefoot running pattern.

The trajectories of 50 reflective markers were recorded using 12 camera system (Vicon Vero 2.2, Oxford Metrics, United Kingdom), sampling at 250 Hz. The markers were placed at specific anatomical position on the subjects’ head, trunk, arms, pelvis, lower limbs and foots. This marker set was proposed by Lai et al. (2015) to investigate the ankle moment generation. Moreover, we added another 14 markers (five on the right shank/foot and one at the great trochanter and cheekbones, bilaterally) to measure the tendon lever arm during running as described by Rasske et al. (2017) and the internal work (see below) with the marker set proposed by Minetti et al. (1993).

Ground reaction forces (GRFs) were recorded using an instrumented treadmill (M-GAIT, MOTEK) with two 3-axial (horizontal, vertical and mediolateral) force plates sampling at 1500 Hz (Lai et al. 2018). Resultant GRFs, centre of pressure and free moment vectors were measured and recorded by the treadmill’s software.

A B-mode ultrasound apparatus (Telemed Echo Blaster 128) with a linear probe operated at a scanning depth and with of 6 cm (sample frequency 7 MHz) was used to record the GM fascicles at a sampling rate of 60 Hz. Ultrasound images were recorded from the right leg of each athlete during each running trial, with the probe placed in the sagittal plane at the mid-belly of the muscle. The position of the scanning probe was manipulated until the superficial and deep aponeuroses and the connective tissue that surrounds the muscle fascicles were clearly visible (Lichtwark et al. 2007; Cronin and Finni 2013).

All signals were synchronized by a digital output generated by the ultrasound scanner that triggered all instrumentations (the Vicon cameras, ground reaction forces and ultrasound).

A metabolic gas analysis was performed to measure oxygen consumption during each running trial ($\dot{V}$O_2_) by means of a breath by breath metabolimeter (CORTEX Metalyzer 3B, CORTEX Biophysik, Germany). Six min of baseline data collection in a standing position was performed before these tests, the running trials were separated by 5 min of rest and data collected in the last minute of rest/exercise were averaged and used in further analyses. Net energy cost (*C*_net_) was calculated as ($\dot{V}$O_2net_/*v*), by dividing net oxygen uptake ($\dot{V}$O_2net_ = $\dot{V}$O_2_ − $\dot{V}$O_2rest_), in ml O_2_ kg^−1^ min^−1^, with the treadmill velocity (*v*, expressed in m min^−1^) and using an energy equivalent that takes into account the respiratory exchange ratio (RER): $\dot{V}$O_2net_ (4.94·RER + 16.04) J ml O_2_^−1^ (Garby and Astrup 1987); *C*_net_ is thus expressed in J kg^−1^ m^−1^. In previous studies quantifying “apparent” efficiency, net energy expenditure ($\dot{V}$O_2net_ instead of overall $\dot{V}$O_2_) has invariably been utilized to calculate the cost of transport (e.g. Minetti et al. 1993, 2001, 2002; Saibene and Minetti 2003; Zamparo et al. 2002, 2016) because, in addition to the metabolic cost of locomotion, overall $\dot{V}$O_2_ also encompasses resting energy expenditure, which is not “utilized” to transport the body.

### Data analysis

In the last minute of each running trial, kinematic, kinetic and ultrasound data were analysed during the stance phase of ten consecutive steps for each participant. This timing was chosen to coincide with the determination of oxygen uptake. Data of each instrumentation (except for the oxygen consumption data) were interpolated to 200 sample points.

#### Kinetics and mechanical work

Marker trajectories were filtered with a forward and reverse low-pass Butterworth filter (second order: cut-off 10 Hz), whereas GRF was filtered through a forward and reverse low pass, fourth-order Butterworth filter with a cut-off frequency of 30 Hz (consistent with the Nyquist theorem). Spectral analysis showed peaks of noise frequencies at 41, 47 and 100 Hz, which were speed and gait independent and consequently induced by the treadmill engine.

Inverse kinematics was used to calculate the angular rotation for each body segment (Lai et al. 2015, 2018). The foot was modelled as a rigid segment and the ankle joint was represented as a universal joint with the centre of rotation at the midpoint between the medial and lateral malleoli markers and was reconstructed relative to the shank line (Schache et al. 2011). A standard inverse dynamic approach was used to obtain ankle joint torque, while ankle joint power was calculated as the product of ankle joint moment and ankle joint angular velocity.

To calculate the internal work, the body was considered to be composed of 11 body segments: head–trunk, thighs, shanks, feet, upper arms and forearms (Minetti et al. 1993). Based on the intrinsic characteristics of the limbs (mass of each segment and radius of gyration) determined according to Dempster inertial parameters (Winter 1979), and their 3D angular velocity and acceleration, the work necessary to rotate and accelerate the limbs with respect to body centre of mass (BCoM) (e.g. the internal work, *W*_INT_, J kg^−1^ m^−1^) was calculated (Cavagna and Kaneko 1977; Minetti 1988; Minetti et al. 1993; Pavei et al. 2017).

The work done to raise and accelerate the body centre of mass with respect to the environment (*W*_EXT,_ J kg^−1^ m^−1^) was calculated based on the summation of all increases in total mechanical energy (*E*_T_ = *E*_P_ + *E*_K_), where the time course of potential (*E*_P_) and kinetic (*Ek* = *Ek*_*x*_ + *Ek*_*y*_ + *Ek*_*z*_) energy were calculated based on the BCoM trajectory. The BCoM position was calculated by a double integration of the GRF signal, according to Cavagna (1975), and using as integration constant the treadmill speed (as described by Saibene and Minetti 2003).

The sum of internal and external work represents the total mechanical work generated to move the body over a unit distance (*W*_TOT_, J kg^−1^ m^−1^). “Apparent” efficiency (AE) was then calculated from the ratio *W*_TOT_/*C*_net_ (both expressed in J kg^−1^ m^−1^, see above).

Total mechanical work (*W*_TOT_, as computed here) is, therefore, not the total work done by the muscles or by the MTUs but represents the mechanical work at whole-body level. Moreover, by means of this method, instead of the work of a force, the work done on the body is computed; this work, in turn, is calculated based on the work–energy principle, which states that the work done on an object is equal to the change in its (kinetic and potential) energy (e.g. Zatiorsky 2000).

#### Muscle fascicle and series elastic element behaviour

In vivo muscle fascicle length and pennation angle were measured from the ultrasound videos. Pennation angle was defined as the angle between the collagenous tissue and the deep aponeurosis (Lichtwark et al. 2007; Seynnes et al. 2015). A validated automatic tracking algorithm was used to quantify muscle fascicle length and pennation angle (Cronin and Finni 2013; Gillet et al. 2013). Each frame of the tracked muscle fascicle lengths and pennation angles was visually examined to check the algorithm’s accuracy. Whenever the muscle fascicle length or pennation angle was deemed inaccurate, the two points on the aponeuroses defining the muscle fascicles were manually repositioned. The instantaneous MTU length of GM in the stance phase was computed using instantaneous joint angles as proposed by Hawkins and Hull (1990); the instantaneous tendon length was calculated as the difference between the MTU length and the muscle belly length, taking into account the effect of pennation angle (Fukunaga et al. 2001). The behavior of the MTU, the fascicles and the Achilles tendon were investigated during the absorption phase (where net ankle joint power is negative) and the propulsive phase (where net ankle joint power is positive) during stance. The average MTU length, fascicle length and tendon length during the absorption and propulsive phases are reported in Table 1 along with tendon strain (the maximum value during the stance phase) and tendon recoil (the maximum value during the propulsive phase). Fascicle and tendon length velocities were computed by differentiating the lengths of each component with respect to time in the stance phase (Lai et al. 2015, 2018).

**Table 1:** Average muscle–tendon unit (MTU) length, fascicle length and tendon length during the absorption and propulsive phases of ground contact at 10, 13 and 16 km h^−1^

	10 km h^−1^	13 km h^−1^	16 km h^−1^
MTU length			
Absorption phase	47.41 ± 3.4	53.33 ± 3.9**^#^	58.87 ± 4.2***^#^
Propulsive phase	44.32 ± 3.2	42.88 ± 2.7*^#^	40.04 ± 2.8**^#^
Fascicle length			
Absorption phase	4.37 ± 1.01	4.28 ± 0.97*^#^	4.19 ± 0.83**^#^
Propulsive phase	3.99 ± 0.98	3.81 ± 0.98*^#^	3.62 ± 0.98**^#^
Tendon length			
Absorption phase	22.53 ± 2.2	23.78 ± 1.97*^##^	24.51 ± 1.89**^##^
Propulsive phase	21.12 ± 1.9	20.02 ± 2.01*^#^	19.48 ± 1.88**^#^
Tendon strain			
Absorption phase	1.10 ± 0.49	1.35 ± 0.52**^##^	1.62 ± 0.57***^##^
Tendon recoil			
Propulsive phase	0.89 ± 0.55	1.11 ± 0.48**^##^	1.35 ± 0.51***^##^

Tendon strain (the maximum value during the stance phase) and tendon recoil (the maximum value during the propulsive phase) are also reported. Data are means ± SD and are expressed in cm

Significant differences from 10 km h^−1^ (*P < 0.05; **P < 0.01; ***P < 0.001); significant differences between 13 and 16 km h^−1^ (^#^*P* < 0.05; ^##^*P* < 0.01; ^###^*P* < 0.001)

#### Muscle fascicle and tendon mechanical work

**Fig. 1:**
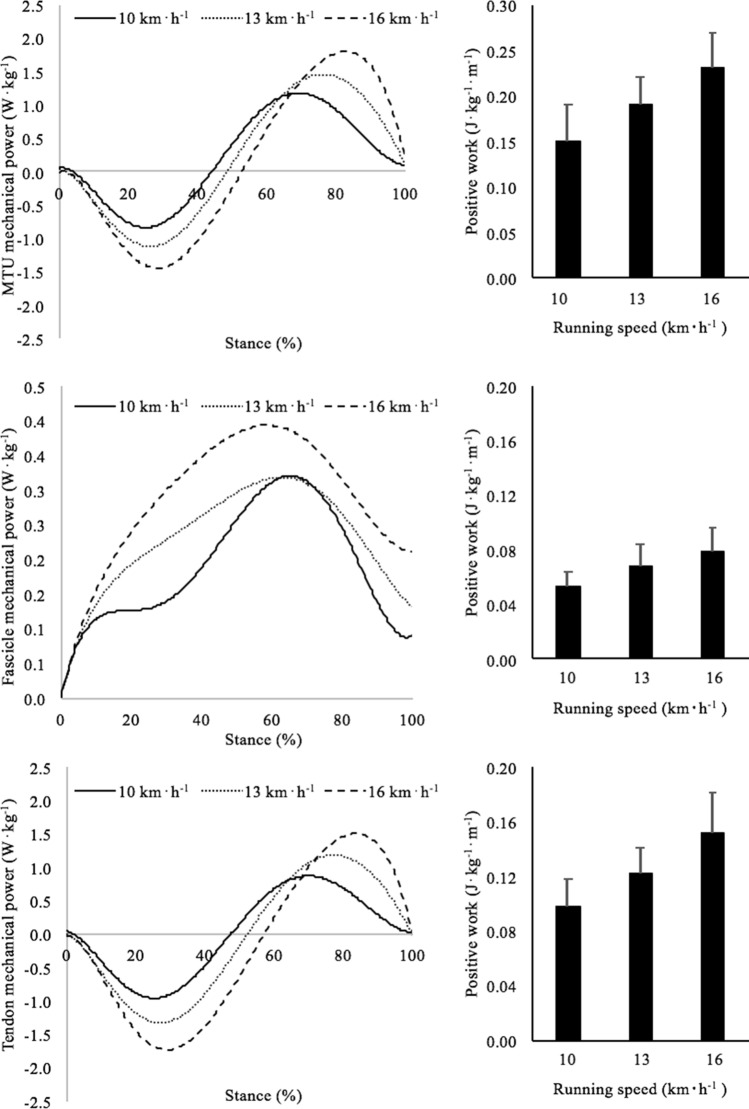
Panels on the left: profile of mechanical power absorbed (negative) and generated (positive) by the MTU (upper panel), muscle fascicle (middle panel) and Achilles tendon (lower panel) during the stance phase at the investigated running speeds (solid line: 10 km h^−1^; dotted line: 13 km h^−1^; dashed line: 16 km h^−1^). Note that the mechanical power absorbed by the Achilles tendon is always higher than that returned during its recoil. Panels on the right: positive mechanical work done by the MTU (upper panel), muscle fascicle (middle panel) and Achilles tendon (lower panel) during the stance phase at all the investigated running speed. Positive work was calculated as the first integral of the positive mechanical power generated during the stance

The amounts of mechanical work done by the MTU, by the muscle fascicles and by the Achilles tendon were calculated by integrating the corresponding power curves over the entire stance phase (see Fig. 1). In turn, the power developed by each component was obtained by multiplying the corresponding force and velocity values. Force production was determined as proposed by Farris and Sawicki (2012) whereas the velocity was calculated as the first derivative of the length changes. Briefly, tendon force was calculated as the net ankle torque divided by the tendon lever arm (estimated as suggested by Rasske et al. (2017). The force attributable to GM was estimated by multiplying “overall” tendon force by the relative PCSA of this muscle which, according to the literature, amounts to ~ 16% of the PCSA of all the plantar flexors (Fukunaga et al. 1996). To estimate muscle fascicle force, tendon force was divided by the cosine of the pennation angle (e.g. Lichtwark and Wilson 2005).

The positive work done by the MTU (*W*_MTU_) was calculated in the portion of stance where the MTU generates positive power (Fig. 1, upper panel). Positive muscle fibre work (*W*_fas_) was calculated as the positive muscle fibre work done during the propulsion phase (Fig. 1, middle panel). From these data, positive tendon work (*W*_ten_) was finally calculated, which represents the mechanical energy that can be derived from tendon recoil during the propulsion phase (Fig. 1, lower panel).

### Statistics

A one-way ANOVA for repeated measures was conducted to test for possible differences among running speeds for all the investigated variables. When significant main effects were found, a post hoc pairwise comparison using Fisher’s least significant difference was used to determine the effect of speed. To determine the relationships between *W*_ten_ and *C*_net_, AE and *W*_TOT_, the Pearson’s correlation coefficient was used. Statistical analysis was performed with SPSS (v24.0). All data extracted for statistical analysis were normally distributed (Shapiro–Wilk normality test, *P* > 0.05).

## Results

Table 1 reports the average values of muscle–tendon unit length, fascicle length and tendon length during the absorption and propulsive phases, as well as the values of tendon strain and recoil. All these parameters increased significantly as a function of speed (main effect: *P* < 0.001), apart from GM fascicle length, which decreased as a function of speed both during absorption and propulsion. Significant differences were observed among speeds in all investigated muscle and tendon parameters.

During the propulsive phase, the average ankle joint power was: 5.40 ± 0.88, 7.38 ± 0.96, 9.72 ± 0.91 W kg^−1^ at 10, 13 and 16 km h^−1^, respectively. Significant differences were observed among all running velocities (main effect: *P* < 0.001).

In Fig. 1, the profile of negative (absorbed) and positive (generated) mechanical power of the MTU (upper panel), muscle fascicles (middle panel) and Achilles tendon (bottom panel) during the stance phase are reported in the panels on the left. The panels on the right depict the average values of positive work during the propulsive phase. All variables increased as a function of speed (MTU: *P* < 0.001; fascicles: *P* < 0.05; tendon: *P* < 0.01) and significant differences were observed among running speeds in all the investigated parameters. The Achilles tendon contributed for the 65% of the MTU work.

Table 2 reports mechanical and metabolic data at the three investigated running speeds. With running speed, W_EXT_ decreased (main effect: *P* < 0.001), whereas $\dot{V}$O_2net_, AE, *W*_INT_ and *W*_TOT_ increased (main effect: *P* < 0.001, for all variables). The comparisons among speeds showed significant differences among running trials for each of these variables. No differences in *C*_net_ were observed as a function of speed.

**Table 2:** Mechanical and metabolic data (mean ± SD) during running at 10, 13 and 16 km⋅h^−1^

	*W*_EXT_ (J kg^−1^ m^−1^)	*W*_INT_ (J kg^−1^ m^−1^)	*W*_TOT_ (J kg^−1^ m^−1^)	$\dot{{\varvec{V}}}$O_2net_ (ml kg^−1^ min^−1^)	*C*_net_ (J kg^−1^ m^−1^)	AE
10 km h^−1^	1.60 ± 0.09	0.31 ± 0.05	1.91 ± 0.04	32.2 ± 5.4	3.98 ± 0.42	0.49 ± 0.03
13 km h^−1^	1.49 ± 0.08^**#^	0.67 ± 0.07^***###^	2.16 ± 0.03^**##^	42.9 ± 4.6^***###^	4.04 ± 0.38	0.53 ± 0.03^**##^
16 km h^−1^	1.33 ± 0.08^***#^	0.95 ± 0.08^***###^	2.28 ± 0.06^***##^	52.6 ± 04.2^***###^	4.08 ± 0.34	0.57 ± 0.05^***##^

*W*_*EXT*_ external mechanical work, *W*_*INT*_ internal mechanical work, *W*_*TOT*_ total mechanical work, $\dot{V}$*O*_*2net*_ net oxygen uptake, *C*_*net*_ energy cost of running, *AE* “apparent” efficiency

Significant difference from 10 km h^−1^ (*P < 0.05; **P < 0.01; ***P < 0.001); significant difference between 13 and 16 km h^−1^(^#^*P* < 0.05; ^##^*P* < 0.01; ^###^*P* < 0.001)

**Fig. 2:**
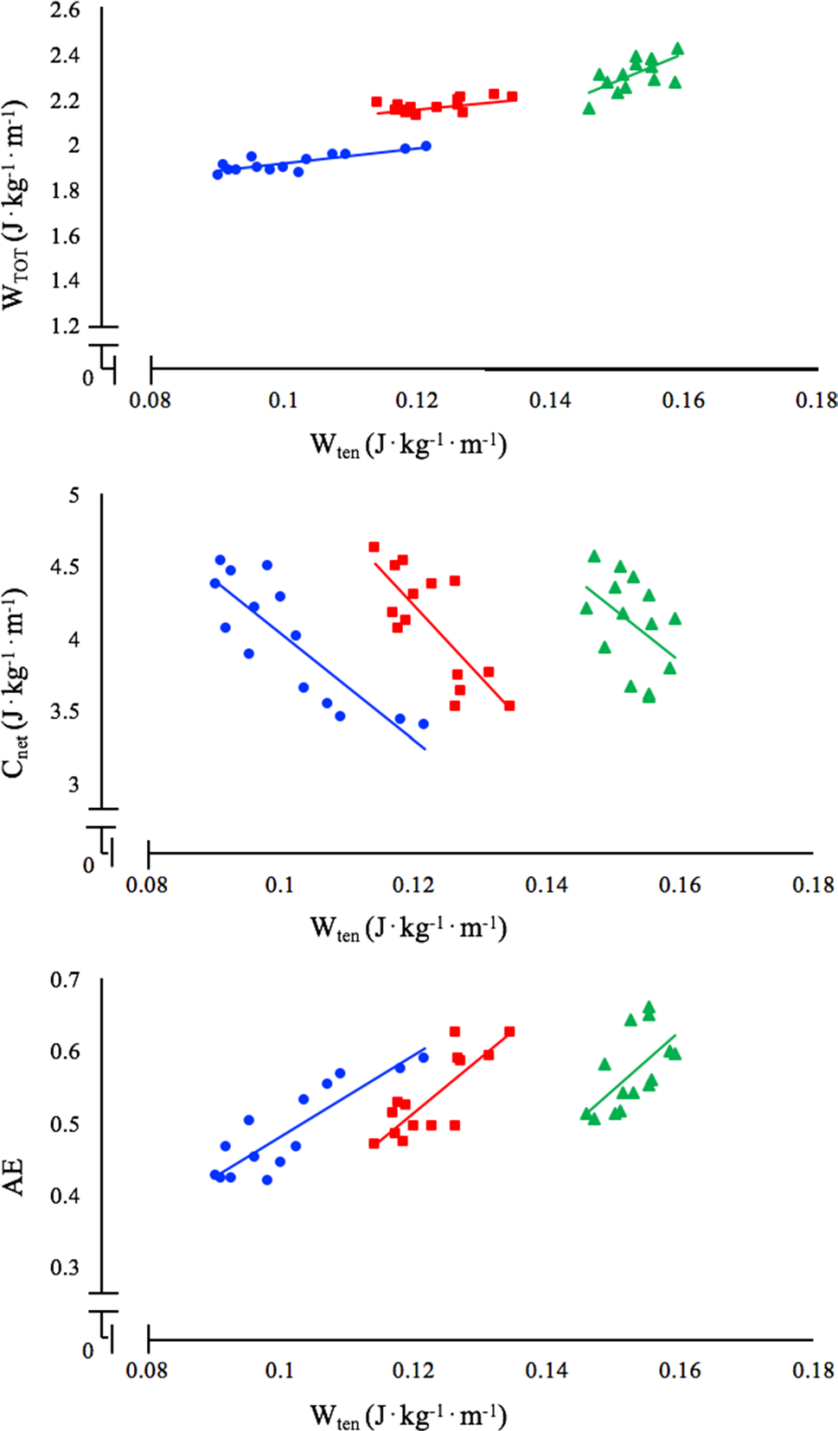
Correlations between (positive) tendon work and total mechanical work (at the whole-body level), net energy cost of running and “apparent” efficiency at the three investigated speeds (blue dots: 10 km h^−1^, red squares: 13 km h^−1^, green triangles: 16 km h^−1^). At each speed, the subjects with the higher tendon work are those with the larger *W*_TOT_, the lower *C*_net_ and the larger AE. Upper panel: correlations between tendon work and total mechanical work at 10 km h^−1^ (*W*_TOT_ = 3.34⋅*W*_ten_ + 1.57, *N* = 15, *R*^2^ = 0.65, *P* < 0.01), 13 km h^−1^ (*W*_TOT_ = 2.82⋅*W*_ten_ + 1.81, *N* = 15, *R*^2^ = 0.52, *P* < 0.05) and 16 km h^−1^ (*W*_TOT_ = 11.40⋅*W*_ten_ + 0.56, *N* = 15, *R*^2^ = 0.55, *P* < 0.05). Middle panel: correlations between tendon work and net energy cost of running at 10 km h^−1^ (*C*_net_ = − 36.56⋅*W*_ten_ + 7.68, *N* = 15, *R*^2^ = 0.72, *P* < 0.001), 13 km h^−1^ (*C*_net_ = − 49.43⋅*W*_ten_ + 10.15, *N* = 15, *R*^2^ = 0.60, *P* < 0.01) and 16 km h^−1^ (*C*_net_ = − 35.36⋅*W*_ten_ + 9.49, *N* = 15, *R*^2^ = 52, *P* < 0.05). Lower panel: correlations between tendon work and “apparent” efficiency at 10 km h^−1^ (AE = 5.61⋅*W*_ten_ – 0.08, *N* = 15, *R*^2^ = 0.75, *P* < 0.001), 13 km h^−1^ (AE = 7.43⋅*W*_ten_ – 0.38, *N* = 15, *R*^2^ = 0.65, *P* < 0.01) and 16 km h^−1^ (AE = 7.96⋅*W*_ten_ – 0.65, *N* = 15, *R*^2^ = 54, *P* < 0.05)

Figure 2 shows the correlations between (positive) Achilles tendon work and *W*_TOT_, *C*_net_ and “apparent” efficiency (AE) at each running speed (10, 13 and 16 km h^−1^, from top to bottom). Significant correlations were observed for all the investigated parameters at all the investigated speeds. The subjects with the highest tendon work were those with the highest total mechanical work (upper panel, *P* < 0.01 in all cases) and with the lower energy cost (middle panel, *P* < 0.01 in all cases). Positive significant correlations were observed between tendon work (*W*_ten_) and AE at all the investigated speeds (lower panel, *P* < 0.001 in all cases): the subjects with the highest tendon work were those with the highest “apparent” efficiency.

**Fig. 3:**
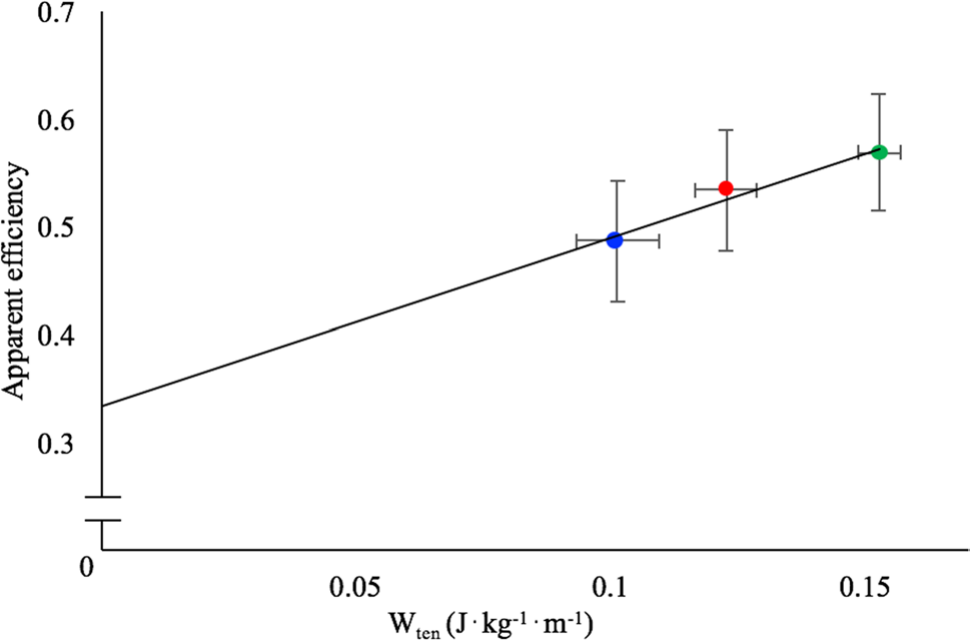
Mean values of “apparent” efficiency as a function of the mean values of (positive) Achilles tendon work, at the three investigated speeds (blue dots: 10 km h^−1^, red dots: 13 km h^−1^, green dots: 16 km h^−1^); this relationship is described by the following equation: AE = 1.56 *W*_ten_ + 0.33. The intercept with the “*Y*” axes (0.33) indicates the value of “apparent” efficiency that could be expected were the Achilles tendon not operating as energy saver

In Fig. 3, the mean values of AE are reported as a function of the mean values of (positive) Achilles tendon work, at the three investigated speeds; this relationship is described by the following equation: AE = 1.56 *W*_ten_ + 0.33.

## Discussion

In this study, we investigated the role of elastic strain energy (e.g. the work produced by the Achilles tendon fascicles of the GM muscle tendon unit) on locomotion (“apparent”) efficiency at increasing running speeds Our results reveal that the work provided by the recoil of the Achilles tendon at each speed is linked to: (1) a reduction in the energy cost of running, (2) an increase in the mechanical work at whole-body level and (3) an increase in the “apparent” efficiency. These novel in vivo experimental results support the notion of elastic energy reutilization impacting positively on the economy/efficiency of running.

### “Apparent” efficiency

Although the contribution of the Achilles tendon to the economy/efficiency of running through the reutilization of elastic energy is a mechanism since long postulated, data reported in this paper constitute a novel observation for in vivo human running, allowing for a better interpretation of the changes in AE in different experimental conditions. In this study, AE was calculated based on values of (total) mechanical work (i.e. the sum of internal and external work). Although the concept of total mechanical work estimation has been debated (as also acknowledged for some aspects by the original authors, Willems et al. 1995), an increase in efficiency attributable to elastic energy reutilization was also observed in studies where mechanical work was calculated based on a different (joint power) approach. As an example, Farris and Sawicki (2011) calculated values of efficiency of about 0.45 during running at 11 km h^−1^ and Voigt et al. (1995) reported values of efficiency of about 0.65 during hopping (with a frequency of 2 Hz). This supports the idea that measuring “apparent” efficiency can help in understanding whether mechanical work has been “recycled” via storage and release of elastic energy, thus indicating the presence of an energy saving mechanism (as suggested by Alexander 1991).

### Muscle and tendon contribution

Regarding the underpinning mechanisms, a possible explanation for the increase in “apparent” efficiency with speed is that the plantar flexor muscles favour the use of tendon elastic strain energy over muscle fibre work (Lichtwark et al. 2007), and that this energy is enhanced when running speed advances towards maximum running velocity (Cavagna and Kaneko 1977). Our data are in line with these considerations and are comparable to those of previous studies that have estimated the relative contribution of tendon elastic strain energy to the positive work done by the MTU for the ankle plantar flexors during running (Hof et al. 2002; Farris and Sawicki 2012; Lai et al. 2014).

This mechanism may be a consequence of the plantar flexor muscle fibres remaining relatively isometric as running speed increases, a behaviour that allows to generate large muscle forces and facilitates the storage and recovery of tendon elastic strain energy. This was recently verified by in vivo studies that analysed the muscle and tendon behaviour of the plantar flexors during running at increasing speed (Lai et al. 2018; Werkhausen et al. 2019; Monte et al. 2020; Bohm et al. 2019). For instance, Monte et al. (2020) demonstrated that the gastrocnemius medialis muscle fascicles shorten during the entire stance phase, but that the series elastic components accommodate much of the displacement of the MTU, allowing the series elastic components to provide the larger amount of mechanical power of the MTU. This result suggests that fibres in distal limb muscles, such as the ankle plantar flexors, act like isometric struts to facilitate greater storage and recovery of tendon elastic strain energy at fast locomotion speeds (as indicated by Biewener and Roberts 2000).

The strong relationship between tendon work and total mechanical work at whole-body level we observed (middle panel of Fig. 2) suggests that the elastic energy provided by the Achilles tendon recoil during the propulsive phase would affect the total mechanical work provided by the body. Indeed, as showed by Monte et al. (2020) with faster running speed, the GM muscle fascicle operating range shifts towards smaller lengths (on the ascending limb of the *F*–*L* relationship) yet operating quasi-isometrically and at a high force potential (> 80% of the maximum isometric force) and this behaviour allows the muscle fascicles to reduce the amount of mechanical work performed. The series elastic components accommodate the largest part of the MTU displacement as they are stretched not only by the muscle force but also, and to a much larger degree, by the ground reaction forces. This allows the AT to perform the largest amount of mechanical work within the MTU.

What is the benefit of a larger tendon work? If the tendon makes a contribution to the whole MTU work during muscle contraction, the contribution of the active contractile components would be reduced, thus reducing the energy expended during the contraction (Roberts and Azizi 2011). In particular, as also reported in the “Introduction”, if the whole MTU length changes were attributable to the tendon alone, the muscle fibres would operate under “pure” isometric conditions and at a high force potential, requiring the lowest level of muscle activation (Fletcher and MacIntosh 2017). This phenomenon was recently verified by Bohm et al. (2019), who observed that the subjects with the higher soleus muscle force potential were those with the lower energy cost of running, suggesting that, the lower the shortening velocity of the soleus muscle the lower the energy demand during running. Furthermore, these authors suggested that the main mechanism for the underlying reduction of the fascicle shortening velocity during the stance phase was a greater tendon gearing (i.e. larger tendon displacement with respect to the muscle fascicles).

However, the utilization of tendon elasticity does not come entirely “free-of-charge”. Tendons operate in series with muscles and can only act as useful springs when muscles generate force. Force generation by muscles requires metabolic energy, and thus there is a cost to operate tendon springs (e.g. Fletcher and MacIntosh 2017; Roberts and Azizi 2011; Roberts 2002). It has been proposed that the net metabolic benefit of tendon elasticity in running is best understood in the context of two properties of skeletal muscle (Roberts 2002; Roberts and Scales 2002). The first is the ‘Fenn effect’, which states that active muscles use more energy when performing work than when generating force isometrically (Fenn 1924). Thus, to the extent that tendons allow muscles to generate force without doing work (or while doing less work), they reduce the rate of energy consumption in the muscle. The second mechanism is the influence that tendon mechanics can have on the recruited muscle volume during running. Owing to the F–L and F–V properties of muscles, force can be produced with fewer active muscle fibres if the muscle operates at low or zero (i.e. isometric) shortening velocity (Fletcher and MacIntosh 2017). The fascicles length changes observed in this study are probably not sizeable enough to increase the metabolic cost of muscle contraction. Indeed, in vitro evidence showed that although muscle force is reduced at shorter sarcomere lengths and a greater muscle activation is needed to reach a given amount of force, the ATPase rate seems not to differ from the rate at optimal length at least until 0.75 of L0; this suggests that if force potential is > 0.75 then muscle metabolic requirement is not affected, whereas when muscle length is < 0.75, a higher cost of contraction should be expected (Stephenson et al. 1989; Joumaa et al. 2017). As shown by Monte et al. (2020) and Bohm et al. (2019), the GM and soleus muscle fascicle do not operate below 75% of their force potential during running, so the effects of fascicle length changes on muscle energy demands could be considered to be negligible.

### Methodological limitations and considerations

In our study, the Achilles tendon length was not directly measured; instead, we used a geometric model to calculate the deformation of the entire Achilles tendon–aponeuroses complex from ankle and knee joint kinematics. However, this approach might overestimate the contribution of the mechanical work done by the tendon only, as shown by Zelik and Franz (2017). In addition, some recent studies (e.g. Kessler et al. 2020) have reported that using a rigid-body foot model (as done in this study) could lead to an overestimation of ankle joint power, thus affecting Achilles tendon mechanical work estimates. One other limitation is the estimation of GM forces based on reported values of relative PCSA, assuming consistent force contribution at all running speeds and a negligible inter-muscular force transmission between the individual plantar flexor muscles. The former assumption has often been used before (e.g. Kurokawa et al. 2001; Fukunaga et al. 1996; Farris and Sawicki 2012) to distribute forces between synergist muscles, but the validity of the outcome forces needs to be confirmed, especially during dynamic conditions. In support of the latter assumption are the findings of Tijs et al. (2015), who have shown that non-myotendinous forces are likely to have a minimal effect on the overall function of muscles.

Besides the Achilles tendon stretch–recoil, there are several other parameters that could affect energy expenditure as running speed increases and have not been considered in our analysis. For instance, higher activation of other agonist and antagonist muscles in the lower limbs, torso and upper limbs would contribute to the increase in metabolic energy expenditure with increasing speed (Arellano and Kram 2014). Also, elastic mechanisms other than Achilles tendon recoil, such as the arch of the foot, could provide extra energy savings with increasing speed and make the running task less energy demanding. Furthermore, elastic energy could be stored also in the transversal plane of the MTU; indeed, biaxial loading of aponeuroses allows for variation in tendon stiffness and energy storage in a variety of locomotor behaviours (such as running, jumping and landing; e.g. Arellano et al. 2019).

## Conclusion

In conclusion, a larger mechanical work provided by the tendons is expected to reduce the metabolic demands of running and to increase locomotion efficiency. Our data support this notion. The relationship between AE and tendon work supports previous suggestions that a value of “apparent” efficiency close to muscle efficiency values (0.25–0.30) should be expected when no elastic energy can be stored in the tendon; the intercept of the relationship between AE and tendon work (0.33) indeed indicates the value that could be expected in the absence of Achilles tendon stretching–recoiling behaviour. As suggested by Alexander (1991), measuring “apparent” efficiency can help in understanding whether mechanical work is “recycled” via storage and release of elastic energy.

## Abbreviations

AE: Apparent efficiency
BCoM: Body centre of mass
*C*_net_: Net energy cost
*E*_k_: Kinetic energy
*E*_p_: Potential energy
*E*_T_: Total energy
*F*–*L*: Force–length relationship
*F*–*V*: Force–velocity relationship
GM: Gastrocnemius medialis
GRF: Ground reaction force
MTU: Muscle–tendon unit
PCSA: Physiological cross-sectional area
*V*: Velocity
$\dot{V}{\text{O}}_{{{\text{2net}}}}$: Net oxygen uptake
*W*_EXT_: External mechanical work (at the whole-body level)
*W*_fas_: Positive work done by the fascicles
*W*_INT_: Internal mechanical work (at the whole-body level)
*W*_MTU_: Positive work done by the MTU
*W*_ten_: Positive work done by the tendon
*W*_TOT_: Total mechanical work (at the whole body level)
